# Patients’ preference for receiving healthcare services from out-of-area hospitals based on the Analytic Hierarchy Process (AHP): A mixed-methods study

**DOI:** 10.1371/journal.pone.0348888

**Published:** 2026-05-29

**Authors:** Hosain Yasemi, Jamil Sadeghifar, Shahin Nargesi, Morteza Arab-Zozani, Khalil Momeni

**Affiliations:** 1 Department of Health Economics and Management, School of Health, Ilam University of Medical Sciences, Ilam, Iran; 2 Health and Environment Research Center, Ilam University of Medical Sciences, Ilam, Iran; 3 Social Determinants of Health Research Center, Birjand University of Medical Sciences, Birjand, Iran; 4 Department of Health Economics and Management, School of Health, Ilam University of Medical Sciences, Ilam, Iran; 5 Health and Environment Research Center, Ilam University of Medical Sciences, Ilam, Iran; Maragheh University of Medical Sciences, IRAN, ISLAMIC REPUBLIC OF

## Abstract

**Introduction:**

Meeting patients’ needs and expectations is one of the core responsibilities of healthcare delivery organizations in any region. Patients have two options for receiving services; either they go to hospitals outside their area of residence to receive medical services or they are referred by physicians to receive these services.

**Objective:**

The present study aimed to identify and prioritize the factors influencing patients’ preference for obtaining healthcare services from out-of-area hospitals (Ilam Province).

**Methods:**

This mixed study was conducted in two qualitative and quantitative phases in hospitals in Ilam city, 2024. In the qualitative phase, 15 experts and specialists were interviewed; data were collected using an In-depth interview guide and analyzed through a conventional content analysis approach. In the quantitative phase, 25 participants were assessed using a pairwise comparison questionnaire, and data were analyzed using the Analytical Hierarchy Process (AHP) with Expert Choice version 11. In the qualitative phase, Data analysis was conducted through employing the software MAXQDA-12.

**Results:**

In the qualitative phase, the 41 identified subthemes were categorized into four main themes: physician-related factors, patient and family-related factors, service-providing organization–related factors, and sociocultural factors. Findings indicated that delayed payment of physicians’ claims, shortage of medical specialists, insufficient medical infrastructure and equipment, and promotional activities by healthcare centers in neighboring provinces played a key role in patient referral and preference for receiving healthcare services outside the province. According to the AHP results, physician-related factors were the most influential criteria, whereas sociocultural factors were the least significant in shaping patients’ preference for out-of-province hospitals.

**Conclusion:**

Given the high annual referral rates and the substantial number of patients traveling outside the province to obtain medical services, health policymakers and provincial senior managers need to implement effective planning and corrective measures to reduce these referrals. Such measures may include recruiting the required specialists and subspecialists, developing practical strategies to improve retention of experienced physicians, enhancing medical equipment and infrastructure, and applying effective marketing strategies in the healthcare sector.

## 1.  Introduction

The main objective of any health system is to ensure the well-being of the population through the provision of high-quality services, as well as the continuous monitoring and evaluation of these services [1]. Hospitals are the most important component of the health care system and are essential pillars of care and treatment delivery. They consume a significant portion of financial resources, human capital, and medical equipment. The importance of their services is such that no health system can function effectively without their support [2].

In Iran, the distribution of healthcare resources across different regions and provinces is highly unbalanced. High-quality health and medical resources—particularly experienced and specialized physicians—are predominantly concentrated in metropolitan cities such as Tehran, Mashhad, Isfahan, Shiraz, Kermanshah, and Tabriz [3]. Consequently, in the face of such unequal distribution, residents of areas with relatively scarce healthcare resources often have no choice but to travel to larger cities to access high-quality medical services [4].

Meeting patients’ needs and expectations is considered one of the most essential responsibilities of healthcare service providers. Attracting patients to hospitals depends on delivering services whose quality and desirability are consistently maintained over time. Today, patients have become increasingly sensitive when choosing healthcare services compared to the past. Providing services that fall short of expectations gradually undermines the credibility and trust of patients and service recipients. Ultimately, patient dissatisfaction and negative word-of-mouth can result in financial loss and even threaten the survival of the organization [5].

Patients’ choice of a hospital is influenced by various factors, including the reputation of medical specialists, service quality, geographical location, costs, advertising and incentives, physical characteristics such as facility design, employee engagement, and the manner in which clinical processes are carried out [5,6].

However, the quality of services in some hospitals in the country – especially in public facilities – does not correspond to the needs and expectations of patients and their families.. Despite substantial investments in diagnostic and therapeutic interventions and the deployment of advanced technologies, patient dissatisfaction remains considerable. This highlights the necessity of fostering effective competition with the private sector and enhancing efforts to attract more patients [7–9].

The primary goal of any medical traveler is to choose the best destination for receiving high-quality and cost-effective medical care. Nevertheless, the reasons for which medical travelers seek healthcare outside their place of residence have not been thoroughly documented. Accurately identifying the components that shape patients’ preferences for selecting out-of-area hospitals is a prerequisite for designing effective policy interventions aimed at reducing this phenomenon, strengthening regional health systems, improving the quality of local services, increasing patient satisfaction, and alleviating pressure on healthcare facilities in other regions [10,11].

While previous studies have mainly examined the quantitative aspects of this issue, a deep and accurate understanding of patient preferences requires a qualitative approach that can also capture patients’ perspectives, attitudes, and lived experiences. Furthermore, once these factors are identified, they must be prioritized based on their importance and influence so that decision-makers can effectively allocate scarce resources.

Ilam Province, located in western Iran, with a population of approximately 635,235 people, includes 12 hospitals (four specialized and subspecialty public hospitals and two specialized/subspecialty private hospitals in Ilam city, along with six general hospitals in other cities). Despite this considerable healthcare center’s capacity, a substantial number of patients travel or are referred to other provinces for medical care (According to statistics from the MCMC system affiliated with the Vice President of Treatment Affairs at Ilam University of Medical Sciences, approximately 971 people annually visit outside the province to receive medical services). Therefore, the present study is the first to identify and prioritize the factors influencing patients’ preference for seeking treatment in out-of-province healthcare centers in Ilam.

## 2.  Methods

This study was conducted in 2024 using a mixed-methods (qualitative–quantitative) design in two phases to identify the factors influencing patients’ preference for receiving healthcare services outside their place of residence and to prioritize these factors using the Analytical Hierarchy Process (AHP).

The study setting included Ilam University of Medical Sciences, the Deputy of Treatment, provincial hospitals, and patients with a history of seeking medical care in other provinces of Iran.

The study population consisted of medical specialists, managers at the Deputy of Treatment, hospital administrators, hospital affairs officers (admission and discharge), insurance experts, nursing office supervisors, researchers, and patients or their family members who had experience or relevant knowledge regarding the study topic.

### 2.1. Ethical approval

The study was submitted to the Ethics Committee of Ilam University of Medical Sciences and approved under the code IR.MEDILAM.REC.1403.116. An official authorization was then obtained from the Vice-Chancellor for Research and Technology to conduct the interviews.

### 2.2.  Data collection

Data were collected from 24 July 2024 to the end of December 2024. In the first phase, data were collected through in-depth, face-to-face interviews with 15 experts with least four years of managerial, operational, or research experience in healthcare delivery, referral systems, or patient care. Participants were selected through purposive and snowball sampling.

At the beginning of each interview, the purpose of the study was explained to the participant, and the objectives of the interview were clearly outlined. To increase participants’ readiness, interview questions were provided to them in advance to facilitate accurate data collection. Interviews were conducted on a floating basis, over a 30-day period. Informed verbal consent was obtained before each interview. In five cases, interviews were postponed due to participants’ busy schedules, although the interview was rescheduled at their own request.

Each interview was conducted by two interviewers: one led the questioning, while the other took notes and recorded the interview. Each in-depth interview lasted approximately 30–50 minutes. The interview guide was initially developed based on existing literature on current challenges of healthcare centers in Ilam. Additional questions were later incorporated and finalized after conducting three pilot interviews. All questions were designed to identify factors affecting patients’ preference for selecting healthcare facilities outside the province. The interview questions are listed in Table 1.

**Table 1:** Interview questions.

1. What are the main reasons why patients prefer to choose hospitals outside Ilam city?
2. What challenges do hospitals in Ilam city face in terms of providing medical services?
3. What are the infrastructure problems in private and public hospitals in Ilam city?
4. What is the role of doctors and human resources in Ilam hospitals in referring patients to hospitals outside Ilam city? Explain the reasons
5. What are the management problems in hospitals in Ilam city?
6. Can you explain other factors that make patients prefer to choose hospitals outside Ilam city?

The researchers concluded that data saturation occurred in the interview with person 13, but two subsequent interviews were conducted for greater certainty.

In the second phase, 25 experts in the fields of health management, referral systems, and healthcare policymaking were selected through purposive sampling. These 25 samples mentioned were people who were in any way in contact with patients, including doctors, hospital managers, admission and discharge experts, treatment managers, etc. The factors derived from the qualitative phase were used to design a pairwise comparison questionnaire consisting of four main domains. The questionnaire was developed so that each factor could be evaluated against all others through pairwise comparisons. A total of 30 questionnaires were distributed via email to experts, and 25 were completed and returned.

Participants were instructed to compare each factor with the others and indicate the degree of its importance. This evaluation was performed using the AHP scale, in which numerical values represented relative importance: extremely important (9), very important (7), more important (5), slightly more important (3), and equally important (1). Experts assigned the appropriate value for each pairwise comparison. The Consistency Ratio(CR) will be calculated for pairwise comparisons among the factors. In the AHP method, a consistency ratio of less than 0.1 will be considered as a desirable and acceptable level [12]. Content validity of the questionnaire was confirmed by five faculty members and specialists in health policy and healthcare management. All participants provided verbal informed consent before participation.

### 2.3.  Data analysis

In the first phase of the study, the interviews were transcribed verbatim to prepare the textual data for content analysis. The initial content analysis was conducted through a systematic review of the transcribed interviews and field notes [13]. Meaning units were identified in the form of sentences from the interview texts, and initial codes or open codes were extracted from them. After several rounds of careful review, Subthemes were identified by the researchers and entered into MAXQDA version 12.

In the next step, main and sub-themes emerged. Two researchers independently reviewed the interview transcripts, coded relevant semantic units, and categorized the sub-themes under the appropriate main themes in MAXQDA-12. In cases where there was disagreement between the two coders about the placement of specific sub-themes, a third referee was consulted to provide an impartial judgment. To further ensure completeness and to identify any additional subthemes that may not have been captured during the initial rounds of coding, the researchers re-examined the full set of transcribed interviews. This iterative and collaborative analytic process enhanced the rigor and trustworthiness of the qualitative findings.

To ensure the content validity and clarity of the interviews, each audio recording was reviewed immediately after the interview, transcribed verbatim, and then returned to the interviewee for confirmation.

In the second phase of the study, the data obtained from the first phase were analyzed using Expert Choice 11 software based on the Analytical Hierarchy Process (AHP). The inconsistency ratio of the pairwise comparison matrix was examined, and finally, the identified factors were ranked and interpreted according to their calculated relative weights.

## 3.  Results

The interview participants were 15 people working in the public, private, and other sectors. The participants in this study were very diverse in terms of position. Table 2 presents the characteristics of the participants.

**Table 2:** Characteristics of the participants.

Organization type	Participant’s position in the organization	Participant Number
Public	Hospital chief executive	2
	Specialist doctors (Ophthalmologist, Pediatrician)	2
	Treatment Monitoring Manager	1
	Nursing office manager	1
	Hospital receptionist	1
	Hospital discharge officer	1
Private	Hospital chief executive	1
	Specialist doctors (Cardiologist, Orthopedic)	2
	Insurance expert	1
Other	Patient & Patient’s family	2
	Researcher	1
total		15

In the first phase (qualitative results) of the study, analysis and classification led to the identification of four main themes and 41 sub-themes. The main themes included: physician-related factors, patient and family-related factors, service-providing organization–related factors, and sociocultural factors, as summarized in Table 3.

**Table 3:** Themes and Sub-Themes Affecting Patients’ Preference for Receiving Medical Services from Hospitals Outside Ilam Province.

Main Themes	Subthemes	Frequency
**Physician-related factors**	Delayed payment of physicians’ claims	14
	Low salaries and incentives for physicians	13
	Physicians’ fear of treatment outcomes and patient complaints	10
	Inexperienced or temporary physicians	10
	Short tenure of physicians	9
	Shortage of specialists and subspecialists in the province	8
	Physicians’ lack of trust in the provincial healthcare system	8
	Low motivation of physicians	7
	Requests for under-the-table payments	5
	Patronage and collusion with hospitals outside the province	4
	Physicians’ fear of reputational damage due to small population	4
**Patient and family-related factors**	Insistence from patients, family, or relatives due to previous negative experiences	12
	Lack of patient choice	8
	Long waiting times for services	8
	Inadequate communication and responsiveness of physicians	7
	Misconceptions regarding the low quality of local healthcare services	6
	Feeling of insufficient treatment	5
	Poor psychological condition of accompanying family members	5
	Low knowledge and awareness of patients and companions	5
**Organization/service-provider-related factors**	Weak or insufficient healthcare infrastructure	15
	Lack of high-quality paraclinical services	14
	Poor hospital hoteling conditions	12
	Outdated or worn-out medical equipment	12
	Inadequate communication about hospital capacities and services	8
	Lack of monitoring of the referral system	8
	Low motivation of nurses and non-physician staff	8
	Delays in insurance payments	7
	Gaps in laws and upstream regulations	7
	Absence of capable private sector	6
	Patient referral from public hospitals to other hospitals by vested interests	5
	Failure of companies to supply medical equipment due to unpaid claims	5
**Sociocultural factors**	Rumor-mongering and gossip in the province	10
	Small size of the province and community	9
	Ethnic and tribal issues	9
	Perfectionism in healthcare services	7
	Lack of competitive environment in the province	7
	Lack of trust in local healthcare professionals’ capabilities	6
	Advertising by physicians in neighboring provinces	6
	Interference of unrelated individuals or organizations in healthcare affairs	5
	Lack of support from provincial authorities for the healthcare system	5
	False information about the provincial healthcare system on social media	4

In the factors related to physicians, the most frequent subthemes are related to “Delayed payment of physicians’ claims” and the least frequent subthemes are related to “Physicians’ fear of reputational damage due to small population”. The most frequent and least frequent sub-themes of other factors are reported in Table 3.

### 3.1.  Physician-related factors

#### 3.1.1.  Delayed payment of physicians’ claims.

One of the key factors driving patients from Ilam to seek healthcare services outside the province is financial issues related to the delayed payment of physicians’ claims. Fourteen interviewees highlighted this problem.

“*If our specialist physicians could receive their fees within one or two months, or even three months, they would be willing to work in the public sector. Sometimes, payments to our physicians are delayed for ten or eleven months, and occasionally more than a year. The physician provides services for a minimum wage, which may only be paid a year later.” (Participant 5)*

#### 3.1.2.  Physicians’ fear of treatment consequences and patient complaints.

Another factor reducing physicians’ risk-taking is the fear of potential complications and complaints. This concern discourages physicians from treating high-risk patients locally, often resulting in referrals to hospitals outside the province.

*“Another issue regarding referrals is that, because the city is small, if a high-risk procedure were performed elsewhere, complications might occur. Physicians in small cities are afraid of potential complaints. They may avoid treating patients they know are high-risk or have comorbidities.” (Participant 10)*

#### 3.1.3.  Low retention of physicians.

Most physicians coming to Ilam for mandatory service under national workforce plans are non-local. After completing their term, they usually leave, and new physicians replace them. This cyclical turnover exacerbates public distrust in the governmental healthcare system.

*“There is also the issue of many inexperienced physicians at the provincial center. Unfortunately, the public trusts them less. After gaining experience, they often move to larger cities. The physician’s term ends, and a new one replaces them—the situation remains the same.” (Participant 1)*

#### 3.1.4.  Under-the-table payments.

Under-the-table payments, often seen as a symbol of irregularities in the healthcare system, had decreased with the implementation of Iran’s Health Transformation Plan. However, following the plan’s phase-out, delays in insurance payments, weak enforcement, and lack of effective oversight have led to a resurgence of this practice.

*“In my opinion, many patients who cannot afford private centers are pressured when the university does not pay physicians on time. Physicians then request informal payments (under-the-table). Some patients cannot pay this extra fee and therefore prefer to go to another province.” (Participant 8)*

### 3.2.  Patient- and family-related factors

#### 3.2.1.  Pressure from patients, families, and relatives due to previous negative experiences.

Hospitals, as service-providing organizations, rely on patients and their families as customers. The quality of services significantly affects whether patients return and whether they promote the hospital positively. Dissatisfaction can lead to negative word-of-mouth, discouraging others from using the hospital’s services.

“*Another issue is the pressure from patients’ families and relatives. Sometimes, unpleasant experiences—such as poor treatment, undesirable outcomes, or inappropriate behavior at local public centers—drive patients and their families to seek care outside the province.” (Participant 13)*

#### 3.2.2.  Lack of knowledge and awareness among patients and their companions.

Insufficient knowledge and awareness among patients and their families can negatively impact treatment processes and patients’ quality of life. This gap may result in improper disease management, non-compliance with medical instructions, and exacerbation of symptoms or complications.

*“As I mentioned, every surgery carries some risks, regardless of where it is performed or who performs it. Even in the most reputable hospitals globally, medical errors exist. Unfortunately, due to a lack of awareness, people are unaware of these realities, leading to distrust towards physicians.” (Participant 3)*

### 3.3.  Organization- or service-provider-related factors

#### 3.3.1.  Inadequate and weak medical infrastructure.

Studies indicate that when healthcare facilities are limited, physicians may be forced to prescribe suboptimal treatments that are not the usual preference or ideal for the patient. This issue is particularly evident in rural or underdeveloped areas, where hospitals may lack resources and are compelled to use alternative or low-cost treatments.

“*Another reason is the lack of some facilities. For example, if we have cerebral or cardiac angiography, many referrals would decrease significantly.” (Participant 11)*

*“Also, some patients requiring surgery need an MRI before or after hospitalization. If private hospitals lack MRI, patients must return to public hospitals, which prompts them to go to Tehran or other cities where all services are available in one place. This shows a lack of infrastructure and equipment locally. Currently, public hospitals do not have nuclear medicine or lithotripsy.” (Participant 9)*

#### 3.3.2.  Outdated and worn-out equipment.

Medical equipment plays a crucial role in delivering quality care. Unfortunately, many devices in Ilam hospitals are outdated and irreparable.

“*The equipment we have has mostly been in use for over thirty years and is outdated. Our diagnostic CT scanners have only 16 slices, whereas other provinces have 64-slice or higher machines.” (Participant 2)*

#### 3.3.3. Lack of oversight on the referral system.

Insufficient monitoring of referrals has resulted in uncontrolled or inappropriate patient referrals.

“*Another issue is that there is no supervision over referrals. Which physician has ever been reprimanded for unnecessary referrals, or rewarded for minimal referrals? The university fails to monitor or investigate the reasons for referrals and transfers.” (Participant 15)*

### 3.4.  Socio-cultural factors

#### 3.4.1.  Ethnic and tribal issues.

Cultural and tribal affiliations influence patients’ preferences. Residents of counties with cultural or linguistic ties to neighboring provinces tend to seek care in those provinces rather than the provincial capital.

*“One factor is the cultural and historical affiliations of this province. For instance, northern Ilam counties like Eyvan and Shirvan culturally align with Kermanshah. Patients often prefer to go there for services, even though Ilam’s centers have improved significantly and provide high-quality, affordable care over the past decade.” (Participant 4)*

#### 3.4.2.  Misleading advertising on social media regarding the provincial health system.

Misleading information on social media may exaggerate or distort the quality of medical services and influence patients’ decisions.

*“Social media can either improve or degrade the healthcare system. In recent years, despite awareness efforts, the public perception of Ilam University of Medical Sciences has declined. People believe the services are not acceptable.” (Participant 7)*

*“Social media campaigns often target university officials, claiming appointments are politically motivated rather than merit-based, undermining trust in local healthcare services.” (Participant 6)*

In the second stage of the study, the four main themes, which were explained in detail above, were selected as the main AHP variables and prioritized based on the analytic hierarchy process and pairwise comparisons. Among the four factors, the highest weight or priority was related to physician-related factors with a significance of 0.578, and the lowest importance or priority was related to cultural and social factors with a significance of 0.05, as shown in Table 4.

**Table 4:** Weight/Importance of Each Factor According to Participants’ Perspective.

Factor (Whole Requirements)	Symbol	(Factor Loading)
Physician-related factors	Q1	0.578
Organization/Service-provider-related factors	Q2	0.295
Patient- and family-related factors	Q3	0.077
Socio-cultural factors	Q4	0.050

**Fig 1:**
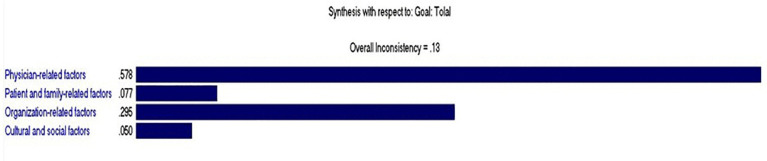
Prioritization of Factors Affecting Patients’ Preference for Receiving Medical Services from Hospitals Outside Their Place of Residence‌‌.

Fig 1 illustrates the prioritization of the factors influencing patients’ preference for seeking care outside Ilam. The chart is based on the weights derived from the Analytic Hierarchy Process (AHP) using Expert Choice software. It clearly shows which categories of factors have the greatest impact on patients’ decision-making.

The Consistency Ratio (CR) for the pairwise comparisons among the factors was calculated as 0.13. In AHP, a CR below 0.10 is considered acceptable and indicates a high level of consistency in judgments. A CR of 0.13 suggests that the participants’ evaluations were somewhat inconsistent in some pairwise comparisons. Nevertheless, this level can still be used for exploratory analysis, particularly in studies dealing with subjective judgments and social factors.

## 4.  Discussion

In this study, the most important factors influencing patients’ preference for receiving medical services from hospitals outside their place of residence were identified and prioritized. The findings indicated that these factors could be categorized into four main themes: physician-related factors, organization-related factors, patient- and family-related factors, and socio-cultural factors.

Low medical tariffs, particularly for experienced physicians with private practices in the public sector, have led many doctors to be reluctant to cooperate with public hospitals, which in turn contributes to patients seeking care outside the public sector and the province. In the study by Mousavi Raja et al., factors affecting physician retention in underprivileged areas included salary levels, benefits, and payment timing [2]. Aatefi et al. reported that poor payment conditions, inadequate salaries, and long working hours were among the main reasons for physicians’ low retention [3]. Farrokhani et al. identified low retention of family physicians at Mashhad University of Medical Sciences in 2015 as being associated with short contract durations, low payment amounts, and the influence of performance evaluation scores on physicians’ salaries [4]. Malayi et al. demonstrated that financial incentives aligned with physicians’ expectations, along with individual and professional support, could enhance their willingness to remain in underserved areas [14–16].

Overall, the evidence suggests that addressing physicians’ financial challenges—particularly regarding medical tariffs—ensuring timely payment of salaries, and providing adequate incentives can increase their motivation to work in the public sector and underserved regions. Consequently, the development of equitable payment policies and the implementation of effective compensation systems are essential for improving the quality and continuity of healthcare services in the country.

Another major challenge in the province’s healthcare system is the shortage of specialists, particularly subspecialists. The lack of subspecialists has led to the referral and transfer of many patients to hospitals outside the province. This issue has widespread implications for the quality of healthcare services, access to specialized medical care, and patient satisfaction. Factors contributing to the shortage of specialists and subspecialists include the concentration of physicians in large urban centers, low attractiveness of deprived areas for physicians, limited educational capacity, and increasing demand for specialized services. This concentration results in an imbalanced distribution of specialized personnel, leaving underserved regions with significant shortages. In some specialties, over 50% of physicians are based in Tehran and other major cities [5]. Underserved areas often lack adequate healthcare infrastructure, welfare facilities, and opportunities for professional development, which further reduces physicians’ motivation to work in these regions and exacerbates the shortage of specialists. Limited capacity of universities and teaching hospitals to train specialists and subspecialists is another key factor contributing to this shortage [6–8]. The implementation of a referral system and family physician programs can reduce the burden on specialists, improve patient access to primary care, and facilitate better human resource management [9].

The existence of some informal interactions and agreements between physicians within the province and their colleagues in other provinces was one of the important issues raised during the study. In some cases, physicians have established connections with their former specialty instructors, classmates, or other active physicians in out-of-province healthcare centers, resulting in patient referrals to these facilities. Such referrals are sometimes motivated by personal benefits, financial gains, or commissions.

Additionally, issues of favoritism and non-transparent relationships with certain out-of-province hospitals were reported during the interviews. In these cases, some physicians refer patients—either through their private practices or even via the official referral system—to out-of-province centers in exchange for special privileges. This practice not only imposes additional costs on patients and the healthcare system but also undermines public trust in the province’s healthcare structure.

In the present study, a direct relationship was observed between patients’ healthcare-seeking behavior and the knowledge, credibility, and reputation of physicians. Given that medical science is inherently empirical and continuously updated with new information and data—sometimes complementing or even contradicting previous findings— physicians need to maintain up-to-date knowledge. Moreover, a physician’s attitude toward professional commitment and the importance of staying current with scientific evidence plays a critical role in the quality of healthcare services. Therefore, to prevent knowledge decay among physicians, emphasis on continuous education, access to reliable scientific resources, and the promotion of a professional responsibility-oriented mindset are essential [17].

Alongside these factors, safety is also a major concern for healthcare personnel, particularly in underserved regions. Working in unfamiliar environments, especially for female medical staff, can reduce motivation. In many cases, mandatory service in such areas without consideration of environmental and security conditions may create a defensive stance in personnel, leading to decreased organizational commitment. Additionally, perceptions of social insecurity further exacerbate this lack of motivation [10].

Illness or accidents resulting in injury impose a heavy psychological burden on patients and their relatives. Naturally, families and close contacts make every effort to safeguard the patient’s well-being. Furthermore, the present study indicates that another factor influencing patients’ and families’ preference for larger healthcare centers is concern about negative judgments or criticism from relatives regarding perceived shortcomings in patient care. Such concerns can have significant psychological effects on caregivers, potentially resulting in guilt, stress, and even occupational burnout [18,19].

Due to pre-existing concerns and cognitive biases, decision-making is often influenced by emotions, leading families to insist on transferring patients to hospitals outside the province. This insistence on referral to larger urban hospitals stems from multiple factors, including greater trust in the quality of care, access to more advanced medical specialties, modern equipment, and previous positive experiences. Kazemi and Hekaren (2012) examined the referral system in rural family physicians and insurance programs in northern Iran. Their results showed that only 33% of referred patients had a referral form from health homes, and 31% of referrals were based solely on the family physician’s judgment. These findings highlight challenges in the effective implementation of the referral system, which may be influenced by patients’ and families’ preference for direct access to major hospitals [20].

In many countries, the referral system serves as a bridge between healthcare providers and recipients, aiming to reduce costs, promote equity, and improve access to necessary services. However, the successful implementation of such a system requires awareness and cooperation from patients and their families to prevent unnecessary visits to specialized centers in major cities. Accordingly, educating and informing patients and families about the benefits of the referral system and its role in enhancing the quality of healthcare services can play a significant role in reducing their insistence on direct visits to urban hospitals [21].

Another influential factor is effective communication between physicians and patients, particularly in interactions with patients’ companions, which is fundamental to improving healthcare quality and increasing patient satisfaction. Numerous studies have demonstrated that inadequate communication and insufficient responsiveness from physicians can lead to a range of negative outcomes. For instance, a study by Kazemi et al. (2019) revealed that in many cases, the physician–patient relationship was unidirectional and based on authority and self-interest, which may contribute to patient dissatisfaction and an increase in medical complaints [11]. Furthermore, physicians’ communication skills directly impact patient satisfaction. Deficiencies in communication during history-taking can negatively affect the quality of healthcare services [22].

Therefore, strengthening physicians’ communication and responsiveness skills when interacting with patients and their companions is essential. Such improvements can enhance the quality of care, increase patient satisfaction, and reduce medical complaints.

One of the major challenges in the healthcare systems of many less-developed provinces, and a key reason for patients’ preference to seek care in hospitals outside their home province, is the lack of medical infrastructure, equipment, and capable private-sector providers offering high-quality services. Various studies have shown that in the absence of adequate healthcare facilities, physicians may be forced to prescribe treatments that are not the preferred options or are not ideal for patients under normal conditions [23].

Improving the quality of medical services remains a core component of hospital development, which can be facilitated through better medical equipment.

Patients who require medical services naturally seek high-quality and reliable healthcare centers [24].

According to existing studies, the use of private healthcare varies depending on the type and severity of medical conditions. Individuals with severe acute conditions often choose private physicians, whereas those with chronic illnesses tend to rely more on public healthcare services [14].

Multiple factors can influence patients to seek care outside their residence. Cultural and social factors, such as recommendations from acquaintances, verbal endorsements from family and friends, and physician advice, play an important role [25,26]. Many studies emphasize the significance of word-of-mouth in the selection of healthcare centers. Public expectations are shaped by verbal recommendations, and dissatisfaction with services among patients or their companions can result in negative word-of-mouth regarding a hospital or even a private clinic. Past negative experiences can profoundly influence behavior and attitudes, leading to psychological stress among family members and companions. In general, adverse past experiences can create psychological pressure and undesirable behaviors in families and caregivers [27].

Furthermore, the literature identifies that quality of care, long waiting times, and lack of access to treatment in the patient’s residence are fundamental factors driving patients toward alternative healthcare destinations. People prefer destinations that provide specialized services related to their health conditions.

Although medical tourism has been explored in various studies, very few investigations in Iran have focused on identifying and prioritizing the factors influencing the selection of treatment destinations, physicians, hospitals, and the referral of patients to healthcare centers outside their home province. Therefore, this study provides valuable insights for policymakers and decision-makers in long-term planning to improve access to alternative options for patients within the healthcare system. Such a strategy can be implemented through expanding medical services relevant to the conditions for which patients travel, as well as through collaboration between the private and public sectors [28,29].

## 5.  Study limitations

One limitation of the study was the inadequate time that specialist physicians could allocate for interviews. Given that a large portion of the participants were actively engaged in healthcare activities, some either canceled their scheduled interviews or could only devote a very short amount of time due to their heavy workload. To address this limitation, the researchers rescheduled interviews to accommodate the participants’ availability.

## 6.  Conclusion

Considering the high annual referral rate from the province and the large number of patients from Ilam seeking medical services in other provinces—which results in increased direct and indirect costs as well as multiple social consequences—policymakers and senior health managers in the province must plan and implement corrective interventions to reduce patient referrals and out-of-province visits.

Based on the prioritization of factors influencing patients’ preferences for seeking healthcare in other provinces, strategic planning for healthcare centers in the provincial capital is warranted. The strategic objectives should include:

a) Recruiting specialist and subspecialist physicians through formal employment (e.g., faculty appointments).
b) Developing retention packages for specialist physicians through incentive programs.
c) Formulating or revising operational guidelines for physician-patient and physician-companion interactions.
d) Strengthening interdepartmental collaboration between the Deputy of Treatment and the University’s Resource Management Development for the enhancement of healthcare infrastructure (e.g., establishing cardiac surgery operating rooms) and strategic acquisition of advanced medical equipment (e.g., CT angiography machines).

These objectives should be incorporated into annual plans and evaluated by the responsible Deputy of Treatment. Marketing and advertising of medical centers in Ilam city to familiarize people with the services provided can be considered as one of the goals in future planning. Ultimately, implementing these measures will address the systemic lack of coordinated efforts to retain patients within the province, which is one of the primary reasons for patients’ preference to seek care in other provinces in the long term.
